# A new survival model based on ferroptosis-related genes for prognostic prediction in clear cell renal cell carcinoma

**DOI:** 10.18632/aging.103553

**Published:** 2020-07-20

**Authors:** Guangzhen Wu, Qifei Wang, Yingkun Xu, Quanlin Li, Liang Cheng

**Affiliations:** 1 Department of Urology, The First Affiliated Hospital of Dalian Medical University, Dalian, China; 2 Department of Urology, Shandong Provincial Hospital, Cheeloo College of Medicine, Shandong University, Jinan, China; 3 Department of Pathology and Laboratory Medicine, Indianapolis, IN 46202, USA; 4 Department of Urology, Indiana University School of Medicine, Indianapolis, IN 46202, USA

**Keywords:** kidney, ferroptosis, pan-cancer biomarker, clear cell renal cell carcinoma, prognosis/survival

## Abstract

In this study, we analyzed the clinical significance of ferroptosis-related genes (FRGs) in 32 cancer types in the GSCA database. We detected a 2-82% mutation rate among 36 FRGs. In clear cell renal cell carcinoma (ccRCC; n=539) tissues from the The Cancer Genome Atlas database, 30 of 36 FRGs were differentially expressed (up- or down-regulated) compared to normal kidney tissues (n=72). Consensus clustering analysis identified two clusters of FRGs based on similar co-expression in ccRCC tissues. We then used LASSO regression analysis to build a new survival model based on five risk-related FRGs (*CARS, NCOA4, FANCD2, HMGCR,* and *SLC7A11*). Receiver operating characteristic curve analysis confirmed good prognostic performance of the new survival model with an area under the curve of 0.73. High *FANCD2, CARS,* and *SLC7A11* expression and low *HMGCR* and *NCOA4* expression were associated with high-risk ccRCC patients. Multivariate analysis showed that risk score, age, stage, and grade were independent risk factors associated with prognosis in ccRCC. These findings demonstrate that this five risk-related FRG-based survival model accurately predicts prognosis in ccRCC patients, and suggest FRGs are potential prognostic biomarkers and therapeutic targets in several cancer types.

## INTRODUCTION

Ferroptosis is a newly discovered form of cell death characterized by iron-dependent lipid peroxidation [1]. Ferroptosis is closely related to metabolism of amino acids, iron and polyunsaturated fatty acids, and biosynthesis of glutathione, phospholipids, NADPH, and coenzyme Q10 [2, 3]. Ferroptosis is inhibited by iron chelators, lipid peroxidation inhibitors, and reduction of intracellular polyunsaturated fatty acids [2]. Preliminary evidence suggests that ferroptosis suppresses tumor growth and progression and is potentially beneficial for cancer therapy [3]. However, the relationship between expression of ferroptosis-related genes (FRGs) and tumorigenesis has not been investigated in detail.

In this study, we systematically analyzed the differential expression and genetic alterations in ferroptosis-related genes (FRGs) in 32 cancer types. We focused on clear cell renal cell carcinoma (ccRCC) for several reasons. The cytoplasm of ccRCC cells is rich in lipids [28].

A recent study showed that aerobic glycolysis was significantly upregulated in ccRCC compared to glioma and lung cancer [4]. The glycolytic metabolites are precursors for the synthesis of fatty acids [28, 29].

Furthermore, aerobic glycolysis, which is an hallmark of cancer cells, is necessary for the robust production of fatty acids that are required for the rapid proliferation and progression of tumor cells [5]. Ferroptosis is also triggered by perturbations in lipid metabolism [3]. Therefore, we analyzed the status of expression of FRGs in ccRCC. We also constructed a new survival model with five risk FRGs using Lasso regression analysis and verified its prognostic significance in ccRCC.

## RESULTS AND DISCUSSION

### Widespread genetic alterations of FRGs in 32 cancer types

**Figure 1:**
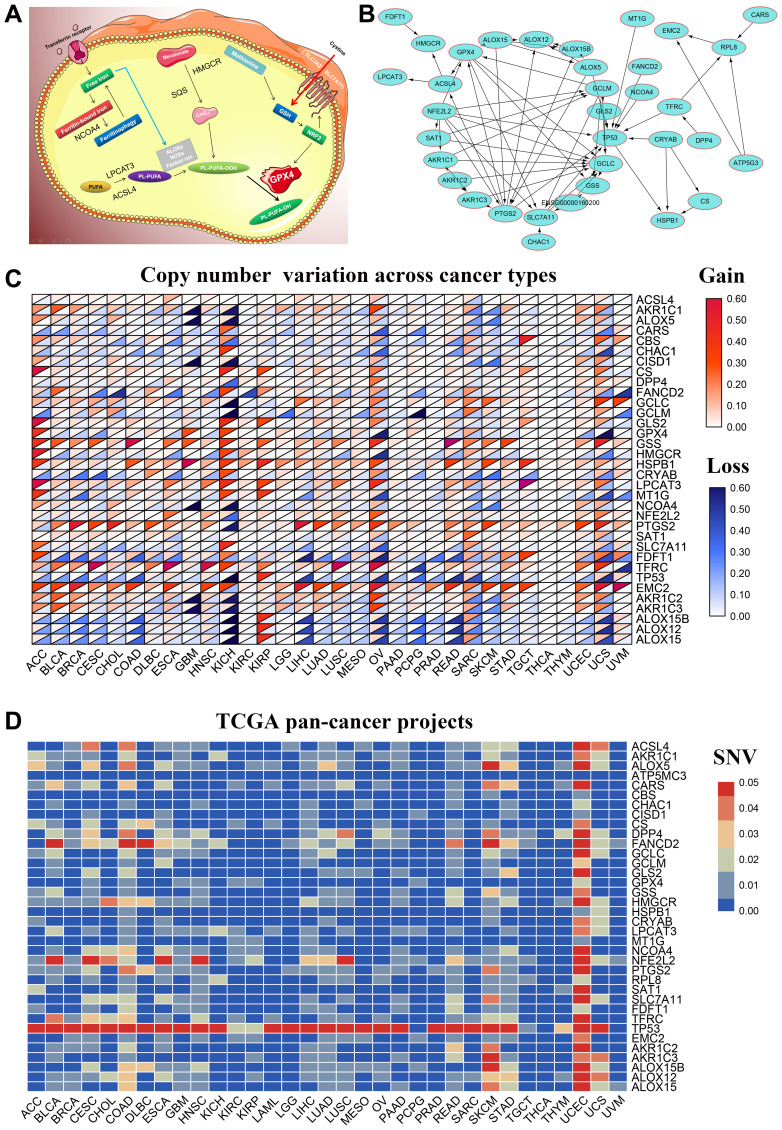
**Genetic alterations and PPI network of 36 FRGs in the TCGA pan-cancer datasets.** (**A**) Diagrammatic representation shows intracellular localization of ferroptosis-related proteins in different signaling pathways. (**B**) The protein-protein interaction network analysis results of 36 ferroptosis-related genes (FRGs) are shown. (**C**) The copy number variation (CNV) frequency of the 36 FRGs is shown for the 32 cancer types. The color code bar on the right refers to differential gain or loss of copy numbers. (**D**) The single nucleotide variation (SNV) frequency of the 36 FRGs is shown for the 32 cancer types. The color code bar on the right refers to differential SNV frequencies.

We performed a comprehensive literature survey [1–3, 6–16] and identified 36 key ferroptosis-related proteins (Figure 1A). Figure 1B shows the protein-protein interactions (PPI) network analysis between these 36 ferroptosis-related proteins using the STRING online database (https://string-db.org) and visualized with the Cytoscape software [17]. We then used the GSCA database [18] to determine the single nucleotide variations (SNV) and copy number variations (CNV) in the 36 FRGs in the 32 cancer types. Our analysis revealed that *TP53, NFE2L2, FANCD2, DPP4, ALOX5, PTGS2, ALOX15B, ACSL4, CARS, HMGCR* were the top 10 FRGs with mutation rates ranging from 2–82% (Supplementary Figure 1A). The average mutation rate of *TP53* was the highest among all FRGs at 82%; majority of the genetic aberrations were missense mutations and were more common in lung adenocarcinoma (LUAD) and squamous cell carcinoma(LUSC) (Supplementary Figure 1A, 1B). We also analyzed the CNVs in the FRGs among the 32 cancer types and found heterozygous mutations in *TP53* and *ALOX15B* and heterozygous amplifications in *RPL8* and *PTGS2* (Supplementary Figure 1C). Then, to verify these results, we downloaded the raw CNV and SNV data of the 32 tumors from the TCGA database, analyzed using the Perl and R languages, and visualized the results using TBtools [19]. These results were consistent with those from the GSCALite website (Figure 1C, 1D).

### Prognostic significance of FRGs in various tumors

**Figure 2:**
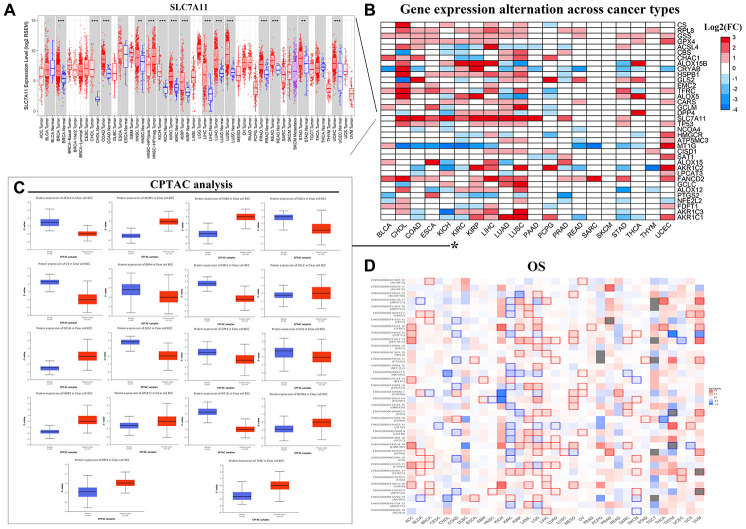
**Pan-cancer mRNA and protein expression of FRGs.** (**A**) Box plots show SLC7A11 mRNA expression in tumor (red) and normal (blue) tissue samples corresponding to 33 cancer types. Note: **P<0.01; ***P<0.001. (**B**) Alterations in the expression of 36 FRGs in 20 different cancer types are shown with the color code bar (right) referring to the corresponding log2 (FC) values. (**C**) Box plots show the differences in the expression of 18 different ferroptosis-related proteins in the KIRC (blue) and normal kidney (orange) tissues from the UALCAN dataset. (**D**) The overall survival of patients belonging to 33 cancer types based on the expression of the 36 FRGs is shown. The color code is shown in the right.

Next, we analyzed the prognostic relevance of FRGs in different tumors. The mRNA expression data analysis of tumor data from the TCGA database for 32 tumors using the R language and TBtools software showed that *SLC7A11*, a representative FRG, was up-regulated in all 32 different tumors compared to the corresponding controls (Figure 2B). Furthermore, TIMER database [20] analysis also showed that SLC7A11gene expression was significantly upregulated in 32 tumor tissues compared to the corresponding normal tissues (Figure 2A). We also analyzed the levels of 18 ferroptosis-related proteins in ccRCC tissues using the UALCAN database [21] and found significant upregulation of ferroptosis-related proteins in the ccRCC tumor tissues compared to the controls (Figure 2C). UALCAN now provides protein expression analysis option using data from Clinical Proteomic Tumor Analysis Consortium (CPTAC) Confirmatory/Discovery dataset. The protein expression for Colon cancer, Breast cancer, Ovarian cancer, Clear cell renal cell carcinoma and Uterine corpus endometrial carcinoma is available [30].

Next, we analyzed the relationship between the expression of FRGs and the overall survival (OS) in 33 different kinds of tumors using the GEPIA online database [22]. The results varied in different tumors. For example, in ccRCC, high expression of *MT1G*, *CHAC1*, and *ALOX5*, as well as low expression of the remaining 33 FRGs correlated with significantly lower OS; conversely, low *ALOX5* expression correlated with reduced OS in Bladder Urothelial Carcinoma (BLCA), Cholangiocarcinoma (CHOL), and Skin Cutaneous Melanoma (SKCM) (Figure 2D).

### Functional analysis of FRG-related pathways in ccRCC

**Figure 3:**
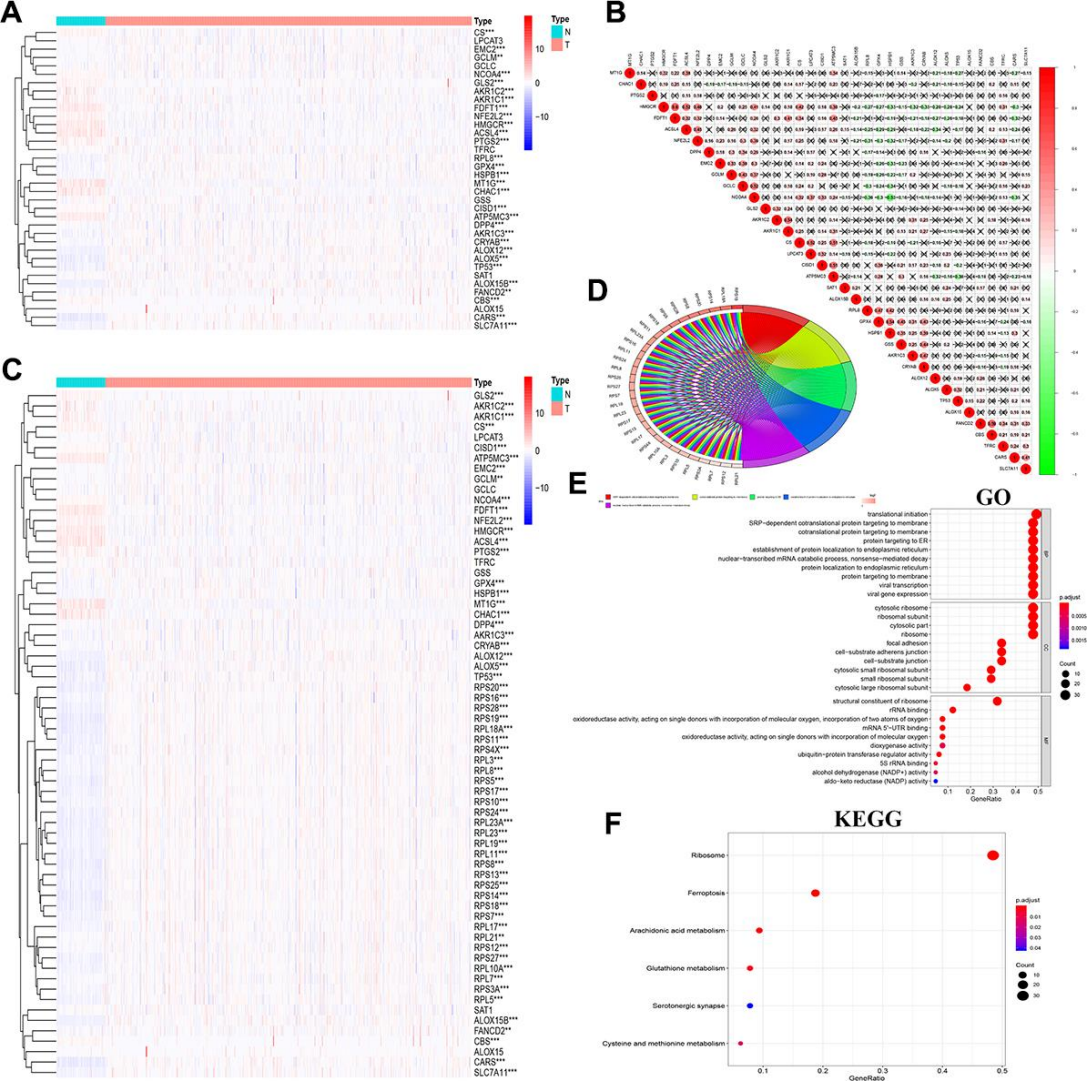
**Correlation and functional pathway analysis of FRGs and FRG-related proteins in ccRCC.** (**A**) The expression of 36 FRGs in ccRCC patient samples is shown. The upregulated FRGs are indicated in red and the downregulated FRGs are shown in blue. N represents tumor sample, T represents normal sample. (**B**) Co-expression analysis shows the correlation between the 36 FRGs based on their expression in ccRCC tissues. (**C**) The expression of 36 FRGs and 30 FRG-interacting proteins in ccRCC is shown with 72 normal kidney tissues and 539 tumor tissues. (**D**, **E**) GO terms representing biological processes for the 66 FRGs and FRG-interacting genes. (**F**) KEGG pathway analysis shows the main signaling pathways represented by the 66 FRGs and FRG-interacting genes. Note: *P < 0.05, **P < 0.01. ***P < 0.001.

The critical role of the Warburg effect and lipid metabolism has been well established in ccRCC [3–5]. Since ferroptosis also involves lipid metabolism, we analyzed the expression of FRGs in 72 normal kidney and 539 ccRCC specimens from the TCGA database use Limma package by R language. The results showed that 30 out of 36 FRGs (Supplementary Table 1) were differentially expressed in ccRCC tissues compared to the normal kidney tissues (Figure 3A). We observed strong correlation among the FRGs, with *GCLC* and *NCOA4* showing a Pearson correlation co-efficient of 0.52 (Figure 3B). Furthermore, we performed gene integration analysis [23–25] to determine the relationship between FRGs and other genes in ccRCC. Protein-protein interaction (PPI) network analysis using the STRING website showed a strong interaction network among the 36 FRG and 30 FRG-related genes (Supplementary Table 2). The heatmap showed that the expression of these 16 genes(MT1G, CHAC1, ACSL, AKR1C2, PTGS2, AKR1C1, CBS, FDFT1, HMGCR, ATP5MC3, GLS2, NFE2L2, CS, NCOA4, CISD1, GSS) in ccRCC tissues was significantly down-regulated and 45 genes (EMC2, RPL21, RPS12, GCLM, RPL7, FANCD2, RPS3A, TP53, RPL5, GPX4, RPS10, RPL3, RPL10A, RPS4X, RPL17, RPS13, RPS17, DPP4, RPL23, AKR1C3, RPL19, RPS7, RPS27, RPS25, RPL8, RPS24, RPL11, RPS16, HSPB1, CARS, RPL23A, RPS11, RPS18, RPS5, RPS28, CRYAB, RPS8, RPS20, RPS14, RPL18A, ALOX12, RPS19, SLC7A11, ALOX5, ALOX15B) was significantly up-regulated compared to the normal kidney tissues (Figure 3C). GO functional analysis of these 66 FRGs and FRG-related genes showed them linked to pathways such as SRP-dependent co-translational protein targeting to membrane, co-translational protein targeting to membrane protein targeting to ER, nuclear-transcribed mRNA catabolic process, and nonsense-mediated decay (Figure 3D, 3E; Supplementary Table 3). KEGG pathway analysis showed that these 66 genes were involved in pathways related to ferroptosis, ribosome metabolism, arachidonic acid metabolism, glutathione metabolism, cysteine and methionine metabolism, and serotonergic synapse (Figure 3F; Supplementary Table 4). Furthermore, we analyzed the GSLA database to determine the role of FRGs in different classical signaling pathways in ccRCC. The results showed that the expression of FRGs was related to the activation or inhibition of multiple oncogenic pathways; for example, FANCD2 expression correlated with the activation of apoptotic, cell cycle, and EMT pathways; AKR1C2 expression correlated with the inhibition of apoptotic and DNA damage response pathways (Supplementary Figure 2).

### Consensus clustering analysis of FRGs reveals two clusters in ccRCC

**Figure 4:**
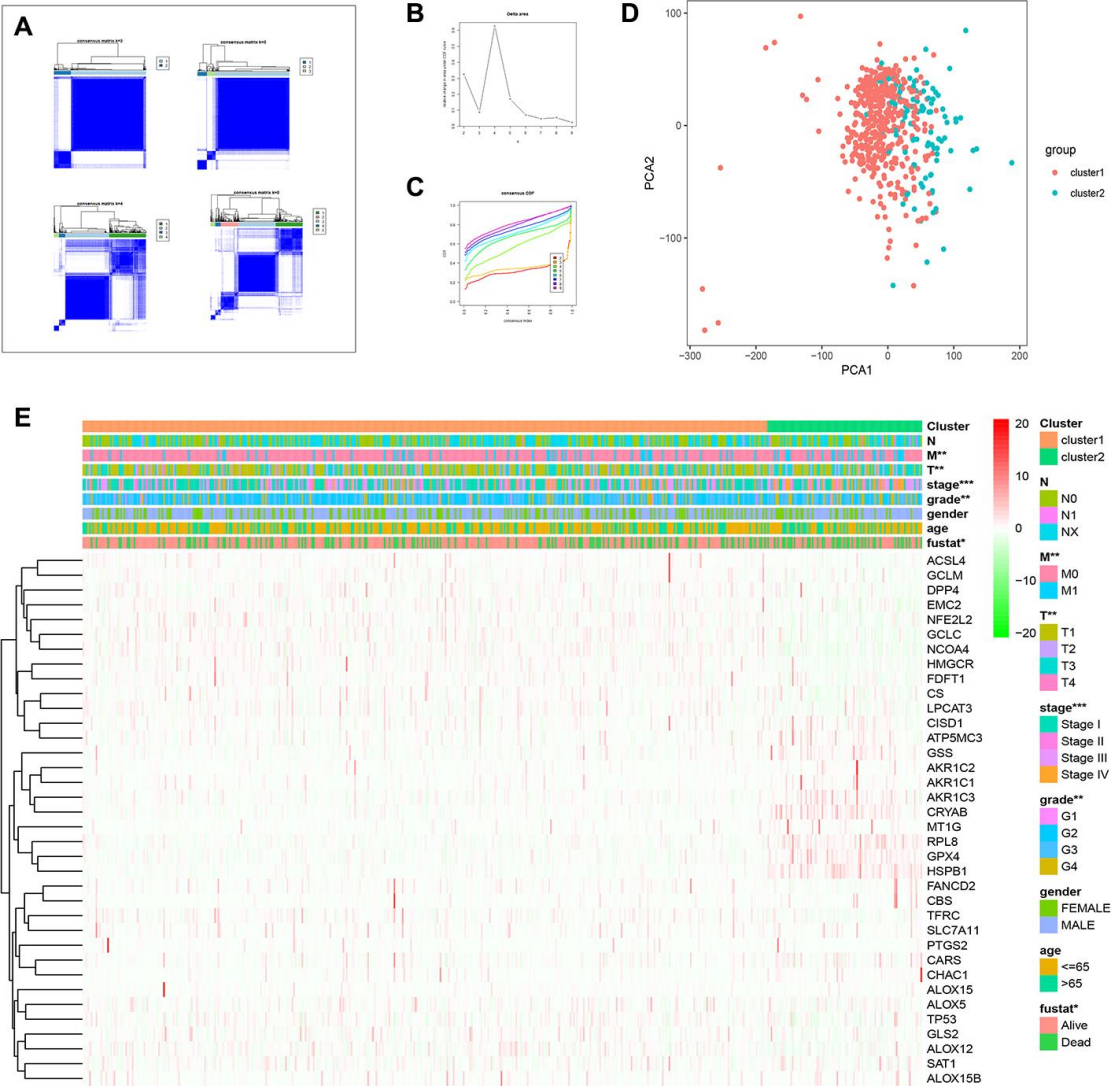
**Consensus clustering analysis of FRGs in ccRCC.** (**A**) The heat maps show the consensus clustering matrix for FRGs in the ccRCC dataset for k = 2, 3, 4 and 5. The optimal clustering is represented by k=2. (**B**) The cumulative distribution function (CDF) plot of consensus clustering matrix for k=2–9 is shown. (**C**) The consensus CDF plots show the cumulative distributive functions of the consensus matrix for k values (indicated by different colors) between 2 and 9. (**D**) Principal component analysis (PCA) of FRGs mRNA expression profiles of the ccRCC patients in the TCGA dataset demonstrates two patient clusters, cluster1 (in red) and cluster2 (in blue). (**E**) Heatmap shows the correlation between the expression of FRGs and the clinicopathological features of the two ccRCC patient clusters, cluster 1 (orange) and cluster 2 (green). The color codes for different clinicopathological parameters are as indicated. The expression of FRGs is also indicated by a color code bar, where red refers to high expression or upregulation and green refers to low expression or downregulation.

Next, we used the commonclusterplus package to identify the different groups of FRGs based on their co-expression patterns in ccRCC tissues from the TCGA database. We divided the FRGs into two groups based on their expression indices using k = 2 as the optimal value because the grouping was suboptimal when they were divided into more than 2 clusters (Figure 4A–4C). The principal component analysis (PCA) confirmed the clustering results of FRGs into two subgroups (Figure 4D). Next, we analyzed the relationship between these two clusters and the clinicopathological characteristics of ccRCC patients. In cluster 1, *RPL8, GPX4, AKR1C3, CISD1, ATP5MC3, GSS*, and *HSPB1* showed significantly lower expression in ccRCC tissues, but the remaining cluster1 genes showed significantly higher expression than the normal kidney tissues (Figure 2E).

On the other hand, the expression of cluster 2 genes correlated with higher tumor grades, stage, and the M- and T-stage (TNM staging) tumors (Figure 2E). Therefore, these results demonstrate that the expression of FRGs is closely related to tumor malignancy and progression in ccRCC patients (Figure 2E).

### Construction and verification of the new FRG-based survival model

**Figure 5:**
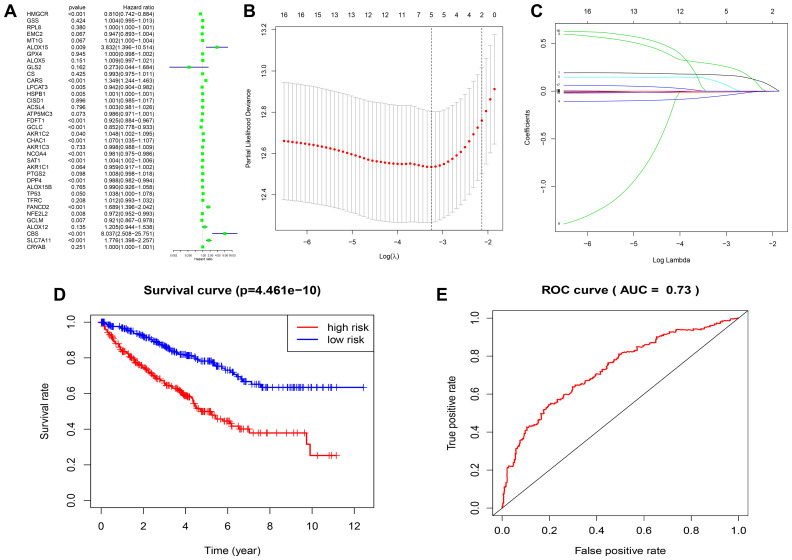
**Construction of FRG-based survival model for prognostic prediction in ccRCC.** (**A**) Univariate Cox regression analysis results show the hazard ratios (HR) with 95% confidence intervals (CI) and p values for the 36 FRGs. (**B**, **C**) Risk score model construction for FRGs using Lasso regression analysis. (**B**) Partial likelihood deviance was plotted against log (lambda). The vertical dotted lines indicate the lambda value with minimum error. The largest lambda value is where the deviation is within one standard error (SE) of the minimum. (**C**) The Lasso coefficient profiles of FRGs in ccRCC. (**D**) Kaplan–Meier survival curves show overall survival of high- and low-risk ccRCC patients that are grouped according to the risk scores calculated by the new survival model based on the expression of 5 FRGs. (**E**) ROC curve analysis shows the prognostic prediction efficiency of the new survival model. As shown, the AUC value for the new survival model is 0.73.

To better understand the prognostic role of FRGs in ccRCC, we performed a univariate Cox regression analysis on the expression of FRGs in the TCGA dataset. The results indicated that high expression of CARS, FANCD2, SLC7A11, CHAC1, SAT1, CBS, ALOX15, and AKR1C2 correlated with worse survival rates in patients with ccRCC. In contrast, high expression of *NCOA4, HMGCR, DPP4, GCLC, FDFT1, LPCAT3, GCLM*, and *NFE2L2* correlated with better survival rates in ccRCC patients (Figure 5A; Supplementary Table 5). We first selected FRGs as survival-related FRGs according to the P-value < 0.05, and then used the LASSO regression model to analyze and determine the most powerful prognostic markers, based on the results, we selected five genes (*CARS, NCOA4, FANCD2, HMGCR, SLC7A11*) to build a risk signature model based on minimum criteria (Figure 5B, 5C). Then, we divided the ccRCC patients into low- and high-risk groups based on the median risk score and investigated the prognostic prediction performance of the new survival model made up of five genetic risk characteristics. Kaplan-Meier survival curve analysis showed that the high-risk group patients had significantly lower survival rates than the low-risk group patients (Figure 5D). Furthermore, we performed ROC curve analysis to analyze the prognostic prediction performance of the new survival model in ccRCC patients and obtained a AUC score of 0.73, thereby demonstrating that the risk score calculated by this model can accurately predict the 5-year survival rate of ccRCC patients (Figure 5E).

### The new FRG-based survival model shows strong association with clinicopathological features of ccRCC patients

**Figure 6:**
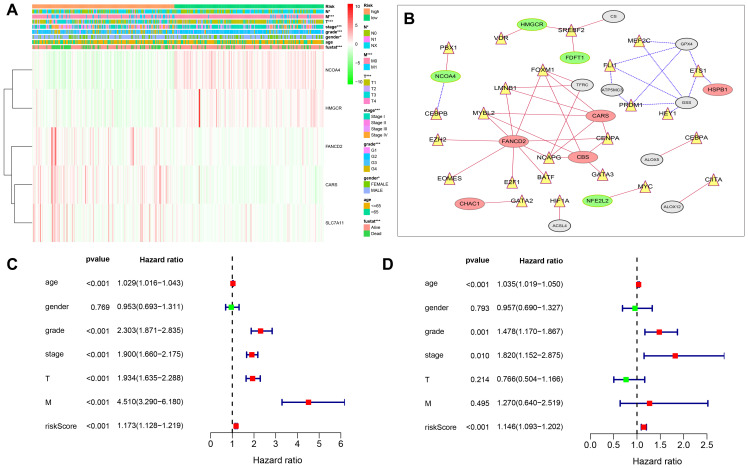
**Relationship between the risk score, clinicopathological features.** (**A**) The heatmap shows the profiles of the expression of survival model FRGs and clinicopathological features in low- and high-risk ccRCC patients. (**B**) Diagrammatic representation shows the regulatory relationship between transcription factors and FRGs. The red lines represent positive regulation, the blue lines represent reverse regulation, the yellow triangle represents transcription factors, the red oval represents up-regulated FRGs, the green oval represents down-regulated FRGs, and gray represents statistically insignificant ERGs. (**C**) Univariate Cox regression analyses results show the association between clinicopathological parameters such as age, gender, grade, tumor size (T), tumor node (N), tumor metastasis (M), and risk score of the new survival model with the OS of ccRCC patients. (**D**) Multivariate Cox regression analyses results show the association between clinicopathological parameters such as age, gender, grade, tumor size (T), tumor node (N), tumor metastasis (M), and risk score of the new survival model with the OS of ccRCC patients. *P < 0.05, **P < 0.01, and ***P < 0.001.

**Figure 7:**
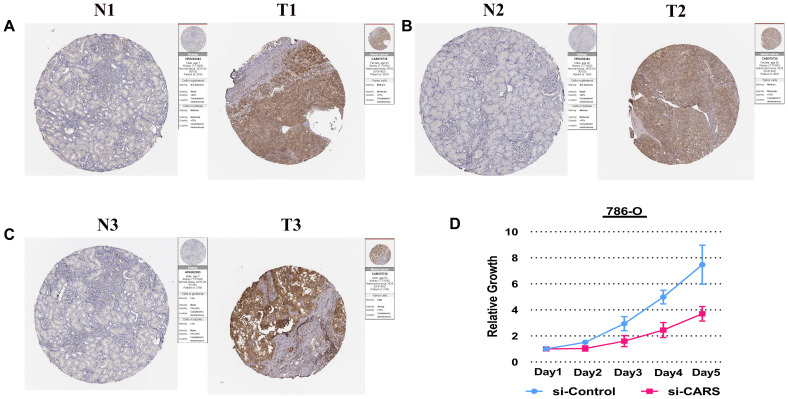
**Experimental verification of CARS.** (**A**–**C**) Immunohistochemical images from the HPA database show CARS protein expression in ccRCC (T) and normal kidney (N) tissues. (**D**) CCK8 assay results show the relative proliferation of si-control- and si-CARS-transfected 786-O cells. The data are shown as means ± S.D.

To better understand the relationship between FRGs and ccRCC, we systematically analyzed correlation between the risk score based on the expression of five FRGs, namely, *FANCD2, HMGCR, SLC7A11, CARS* and *NCOA4* and the clinicopathological characteristics of high- and low-risk ccRCC patients in the TCGA dataset. We observed a strong correlation between the risk score and the clinicopathological characteristics such as T (tumor size), N (tumor node), M (tumor metastasis), tumor grade, tumor staging, gender, and survival in high- and low-risk ccRCC patients (Figure 6A). In the high-risk group, *FANCD2, CARS*, and *SLC7A11* levels were significantly up-regulated, whereas *HNGCR* and *NCOA4* levels were significantly down-regulated (Figure 6A). COX regression analysis showed that risk score, grade, age, tumor stage, tumor size (T), and tumor metastasis (M) correlated with the OS of ccRCC patients (Figure 6C; Supplementary Table 6). Multivariate COX regression analysis showed that risk score, age, stage, and grade were independent risk factors for the prognosis of ccRCC patients (Figure 6D. Supplementary Table 7). Finally, we showed strong correlation between several transcription factors and FRGs and established a regulatory network among them (Figure 6B). Next, we used *CARS* gene to verify our model. The Human Protein Atlas database analysis shows that CARS expression is significantly higher in ccRCC tissues compared to normal kidney tissues Figure 7A–7C [26]. These results were consistent with previous bioinformatics analysis results (Figures 2B, 2C, 3A). CCK8 proliferation assay shows that CARS knockdown 786-O cells showed significant reduction in proliferation compared to the control 786-O cells (Figure 7D). This suggests that CARS may play an oncogenic role in ccRCC, but the specific mechanism needs to be investigated further.

In conclusion, we systematically showed the clinical relevance of FRGs in 32 cancer types. Furthermore, bioinformatics analysis of FRGs in clear cell renal cell carcinoma (ccRCC) demonstrated that the expression of FRGs correlates with several clinicopathological characteristics of ccRCC patients including tumor stage, grade, T- and M-stages. We further constructed and verified a survival model using five FRGs to predict the prognosis of ccRCC patients.

## MATERIALS AND METHODS

### Cell lines, siRNA knockdown, antibodies and reagents

The human ccRCC cell lines 786-O cells were purchased from the Cell Bank of the Chinese Academy of Sciences. All cells were cultured according to the manufacturer's protocol. 786-O cells were cultured in RPMI 1640 medium containing 10% foetal bovine serum, cells were cultured at 37 °C with 5% CO2. cells were transfected with 20 nmol/L siRNAs using Lipofectamine RNAiMAX reagent (Invitrogen).

### Data acquisition and analysis

The SNV and CNV data of 32 cancers was downloaded from The Cancer Genome Atlas (https://cancergenome.nih.gov/) database, analyzed using the Perl language and visualized with the TBtools software. The RNA-seq transcriptome data of the KIRC cohort was downloaded through the R/Bioconductor package TCGAbiolinks with 72 normal kidney tissues and 539 tumor tissues [27] at the Genomic Data Commons (GDC) portal. We also downloaded gene expression, CNV and SNV data for 32 types of cancers as Fragments Per Kilobase of transcript per Million mapped reads (FPKM) at the Genomic Data Commons (GDC) portal. We totally analyzed 32 different TCGA projects, each project represents a specific cancer type, including kidney renal clear cell carcinoma (KIRC); kidney renal papillary cell carcinoma (KIRP); kidney chromophobe (KICH); brain lower grade glioma (LGG); glioblastoma multiforme (GBM); breast cancer (BRCA); lung squamous cell carcinoma (LUSC); lung adenocarcinoma (LUAD); rectum adenocarcinoma (READ); colon adenocarcinoma (COAD); uterine carcinosarcoma (UCS); uterine corpus endometrial carcinoma (UCEC); ovarian serous cystadenocarcinoma (OV); head and neck squamous carcinoma (HNSC); thyroid carcinoma (THCA); prostate adenocarcinoma (PRAD); stomach adenocarcinoma (STAD); skin cutaneous melanoma (SKCM); bladder urothelial carcinoma (BLCA); liver hepatocellular carcinoma (LIHC); cervical squamous cell carcinoma and endocervical adenocarcinoma (CESC); adrenocortical carcinoma (ACC); pheochromocytoma and paraganglioma (PCPG); sarcoma (SARC); pancreatic adenocarcinoma (PAAD); esophageal carcinoma (ESCA); testicular germ cell tumors (TGCT); thymoma(THYM); uveal melanoma (UVM); lymphoid neoplasm diffuse large b-cell lymphoma (DLBC); cholangiocarcinoma (CHOL). The clinical information of cancer patients including information regarding age, survival status, tumor grades, tumor stages, tumor size (T) status, and metastasis (M) status was downloaded from TCGAbiolinks and analyzed with the Perl language and R studio. The expression data of FRGs in 539 ccRCC and 72 normal kidney tissues was analyzed with the Limma package and visualized as a heat map using the TBtools software. Coexpression analysis was performed using the “Corrplot” package. We used the “Consensus Cluster Plus” package to determine the gene clusters, and then used “Ggplot2” and “Limma” package for PCA analysis. We performed LASSO regression analysis with the “Glmnet” and “Survival” packages. The univariate and multivariate Cox hazard analysis of clinical characteristics was performed by "survival" package.

### Establishment of regression model and construction of risk score

After removing the samples without complete clinical information, univariate Cox models were performed to investigate the correlation between the FRGs expression levels and the overall survival (OS) in KIRC patients. We first selected FRGs as survival-related genes according to the P-value < 0.05. Then, Lasso regression was performed to eliminate genes that might overfit the model. Lastly, we applied multivariate analysis to identify the optimal prognostic FRGs for the model. The risk score was calculated based on a linear combination of the Cox coefficient and gene expression. The following calculation formula was used for the analysis: Risk score =Σ ^N^_i=1_ (Expi*Coei). N, Coei, and Expi represented gene number, coefficient value, and level of gene expression, respectively. The median was set as the cut-off value to divided all KIRC patients into low-risk and high-risk groups. Time-dependent receiver operating characteristic (ROC) analysis for overall survival (OS) was used to evaluate the accuracy of the prognostic model.

### Analysis of genome alterations and cellular pathways and GEPIA database

GSCALite (http://bioinfo.life.hust.edu.cn/web/GSCALite/) database was used to analyze SNV and CNV of FRGs in 33 tumors. GSCALite database were also used to analyze the degree of FRGs activation or inhibition of the classical pathway. We used the Gene Expression Profiling Interactive Analysis (GEPIA) database to analyze the OS of patients belonging to 33 tumor types based on the expression of FRGs. cutt-off high value and low value is set to 50%. P <0.05 was considered statistically significant.

### Protein-protein interaction network

Protein-Protein Interaction (PPI) network analysis of DEGs was performed using the STRING database, and functional networks were identified with a medium confidence score of more than 0.4 and other default parameters.

### TIMER and UALCAN analysis

The TIMER online tool was used to analyze the expression of the *SLC7A11* gene in different tumors. The UALCAN online tool is used to analyze the levels of ferroptosis-related proteins in 110 kidney renal clear cell carcinoma (KIRC) and 84 normal kidney tissues. UALCAN now provides KIRC protein expression analysis option using data from CPTAC dataset (84 normal tissues and 110 renal tumor tissues).

### CCK8 cell proliferation assay

We cultured 1×10^3^ 786-O cells per well in 96-well culture plates for 5 days (4 replicate wells per group). Cell Counting Kit 8 (Dojindo, Japan) was used according to the manufacturer’s instructions. Then, we added 10 μL CCK-8 reagent (Dojindo, Japan) to each well and incubated cells for further 1-2 h. Then, we determined the optical density (OD) of each well at 450 nm using a microplate reader.

### Statistical analyses

One-way ANOVA was used to compare the expression of FRGs in tumor and normal tissue samples. The Student’s t-test was used to compare the expression of FRGs in the KIRC dataset according to gender, age, stage, T (tumor size), and M (tumor metastasis) status. N (tumor node) status was not included in the study because it was not verified for a large number of samples in the TCGA database. The cut-off value of each risk score in the tumor group was determined using the “survminer” package, and the patients were divided into high- and low-risk groups according to the best cut-off threshold value. R studio package was used for all statistical analysis. P < 0.05 was considered statistically significant.

## Supplementary Material

Supplementary Figures

Supplementary Table 1

Supplementary Table 2

Supplementary Table 3

Supplementary Table 4

Supplementary Table 5

Supplementary Tables 6-8
