# Supplementary material for: A new survival model based on ferroptosis-related genes for prognostic prediction in clear cell renal cell carcinoma
Source: Aging (Albany NY). 2020 Jul 20;12(14):14933–48. doi: 10.18632/aging.103553 (PMC7425493; doi:10.18632/aging.103553)
Supplement: Supplementary Figures [file aging-12-103553-s007..pdf]

SUPPLEMENTARY FIGURES

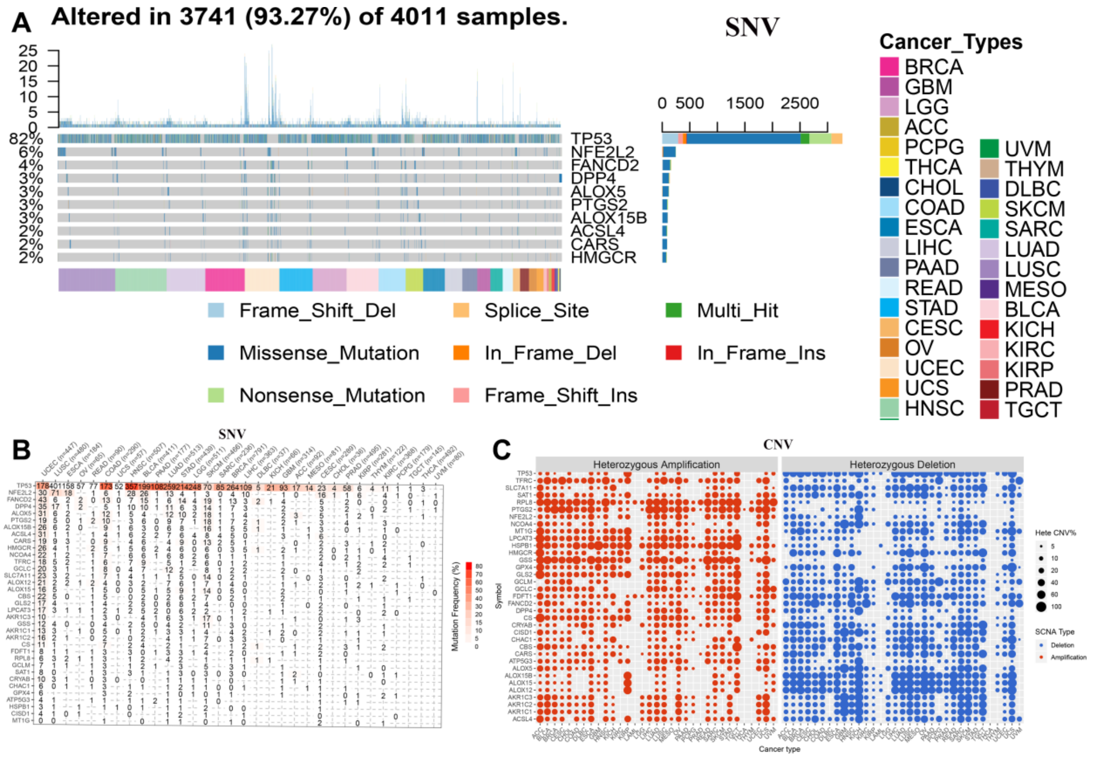

Supplementary Figure 1. Pan-cancer genetic alterations of FRG. (**A**, **B**) The mutation frequency of FRG across 32 cancer types. (**C**) The CNV alteration frequency of FRG across cancer types. The deletion (right) and amplification frequencies (left).

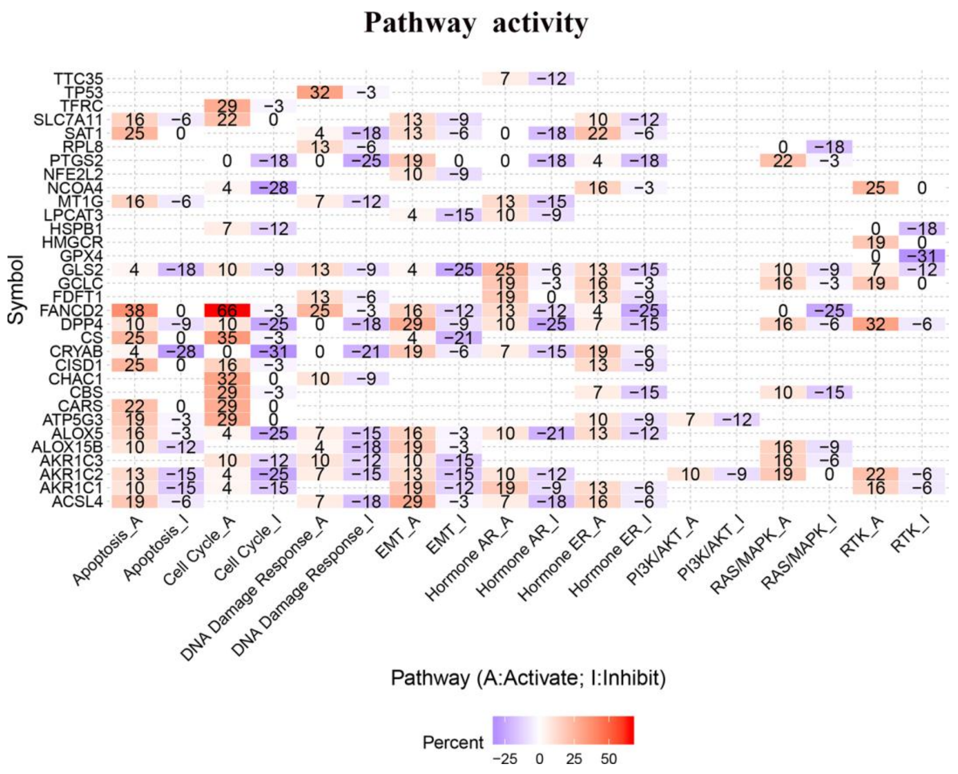

Supplementary Figure 2. Pathway analysis of FRG. Network diagram demonstrating the correlation between FRG and cancer pathways; red = positive correlation, blue = negative correlation.
