# Supplementary material for: A new survival model based on ferroptosis-related genes for prognostic prediction in clear cell renal cell carcinoma
Source: Aging (Albany NY). 2020 Jul 20;12(14):14933–48. doi: 10.18632/aging.103553 (PMC7425493; doi:10.18632/aging.103553)
Supplement: Supplementary Tables 6-8 [file aging-12-103553-s005..pdf]

SUPPLEMENTARY TABLES

Supplementary Table 6. Univariate analysis of risk score and other KIRC clinical data.

id	HR	HR.95L	HR.95H	pvalue
age	1.029049	1.015526	1.042753	2.21E-05
gender	0.953475	0.693387	1.311121	0.769403
grade	2.303129	1.870932	2.835165	3.62E-15
stage	1.900087	1.659546	2.175493	1.48E-20
T	1.934057	1.634871	2.287994	1.44E-14
M	4.509619	3.290467	6.180481	7.51E-21
riskScore	1.172773	1.128116	1.219197	8.55E-16

Supplementary Table 7. Multivariate regression analysis of risk score and other KIRC clinical data.

id	HR	HR.95L	HR.95H	pvalue
age	1.034601	1.019246	1.050187	8.25E-06
gender	0.95717	0.690262	1.327283	0.792975
grade	1.478108	1.170279	1.866907	0.001039
stage	1.819611	1.151655	2.874979	0.010318
T	0.766397	0.503673	1.166164	0.214151
M	1.269594	0.639759	2.519492	0.49485
riskScore	1.14614	1.09283	1.20205	1.99E-08

Supplementary Table 8. Regulatory relationship between transcription factors and FRGs

TF	FRGs	cor	pvalue	Regulation
BATF	FANCD2	0.424352	2.09E-24	postive
CEBPA	ALOX5	0.430824	3.49E-25	postive
CEBPB	NCOA4	-0.40479	3.70E-22	negative
CENPA	CARS	0.444214	7.59E-27	postive
CENPA	FANCD2	0.760034	3.86E-100	postive
CENPA	CBS	0.490092	3.93E-33	postive
CIITA	ALOX12	0.417909	1.19E-23	postive
E2F1	FANCD2	0.584767	1.43E-49	postive
EOMES	FANCD2	0.423908	2.36E-24	postive
ETS1	GSS	-0.47768	2.45E-31	negative
ETS1	GPX4	-0.49883	1.93E-34	negative
ETS1	HSPB1	-0.45975	7.20E-29	negative
EZH2	FANCD2	0.749936	4.24E-96	postive
FLI1	GSS	-0.45969	7.33E-29	negative
FLI1	GPX4	-0.45103	1.01E-27	negative
FLI1	ATP5MC3	-0.43823	4.29E-26	negative
FOXM1	CARS	0.466305	9.38E-30	postive
FOXM1	TFRC	0.407549	1.82E-22	postive
FOXM1	FANCD2	0.695871	2.29E-77	postive
FOXM1	CBS	0.630685	1.10E-59	postive
GATA2	CHAC1	0.43485	1.12E-25	postive
GATA3	CBS	0.52576	1.01E-38	postive
HEY1	GSS	-0.41796	1.18E-23	negative
HIF1A	ACSL4	0.499759	1.39E-34	postive
LMNB1	CARS	0.402936	5.94E-22	postive
LMNB1	TFRC	0.414054	3.33E-23	postive
LMNB1	FANCD2	0.750462	2.64E-96	postive
MEF2C	GSS	-0.44669	3.67E-27	negative
MEF2C	GPX4	-0.45883	9.54E-29	negative
MYBL2	CARS	0.460291	6.10E-29	postive
MYBL2	FANCD2	0.702684	1.72E-79	postive
MYBL2	CBS	0.443459	9.46E-27	postive
MYC	NFE2L2	0.417635	1.28E-23	postive
NCAPG	CARS	0.471307	1.92E-30	postive
NCAPG	TFRC	0.402843	6.09E-22	postive
NCAPG	FANCD2	0.774639	2.36E-106	postive
NCAPG	CBS	0.532948	6.24E-40	postive
PBX1	NCOA4	0.428139	7.36E-25	postive
PRDM1	GSS	-0.42233	3.63E-24	negative
PRDM1	GPX4	-0.43305	1.87E-25	negative
PRDM1	ATP5MC3	-0.43383	1.50E-25	negative
SREBF2	HMGCR	0.56739	3.80E-46	postive
SREBF2	CS	0.504764	2.37E-35	postive
SREBF2	FDFT1	0.492813	1.55E-33	postive
VDR	HMGCR	0.400825	1.02E-21	postive
